# Micronutrient Deficiency and Nutritional Status Among Indonesian Children Under Five Years of Age: Evidence from National Survey Data

**DOI:** 10.3390/nu17243926

**Published:** 2025-12-15

**Authors:** Fitrah Ernawati, Sherry A. Tanumihardjo, Galih Kusuma Aji, Fifi Retiaty, Aya Yuriestia Arifin, Efriwati Efriwati, Dian Sundari, Nunung Nurjanah, Elisa Diana Julianti, Salimar Salimar, Budi Setyawati, Nuri Andarwulan, Noviati Fuada, Muhammad Abshor Dzulhij Rizki, Muhammad Nur Aidi

**Affiliations:** 1 Research Center for Public Health and Nutrition, National Research and Innovation Agency, Cibinong Science Center, Bogor 16911, Indonesia; fifi007@brin.go.id (F.R.); ayay001@brin.go.id (A.Y.A.); efri003@brin.go.id (E.E.); dian079@brin.go.id (D.S.); nunu006@brin.go.id (N.N.); elis007@brin.go.id (E.D.J.); sali005@brin.go.id (S.S.); budi069@brin.go.id (B.S.); novifuada@gmail.com (N.F.); 2 Department of Nutritional Sciences, University of Wisconsin-Madison, Madison, WI 53706, USA; 3 Food Technology Department, Petra Christian University, Surabaya 60236, Indonesia; 4 Research Center for Process Technology, National Research and Innovation Agency, BJ Habibie Science Center, Tangerang Selatan 15314, Indonesia; gali003@brin.go.id; 5 Department of Food Science and Technology, Faculty of Agricultural Technology, IPB University, Bogor 16680, Indonesia; andarwulan@apps.ipb.ac.id; 6 Statistics & Data Science Study Program, School of Data Science, Mathematics, and Informatics, IPB University, Bogor 16680, Indonesia; zidanrizki333@gmail.com (M.A.D.R.); muhammadai@apps.ipb.ac.id (M.N.A.)

**Keywords:** children under five, Indonesia, micronutrients, stunting, wasting

## Abstract

**Background**: Malnutrition and micronutrient deficiencies remain significant public health burdens among Indonesian children under five years of age. This study assessed the relationship between growth indicators and micronutrient status of children aged 0–59 months. **Methods**: A cross-sectional design was performed, utilizing the 2018 Indonesian Basic Health Research (Riskesdas) data and biological specimens, including 550 samples from children aged between 0 and 59 months old. The Riskesdas data used in this study were demographic characteristics, anthropometric measurements, and nutritional status. The biological samples were used to measure micronutrients (iron, zinc, calcium, vitamin A (VA), and vitamin D (VD). **Results**: Overall, 23.1% of children were stunted, and 10.5% were wasted. This study also revealed that the prevalence of micronutrient deficiency was 34.2%, 38.7%, 8.2%, 7.8%, and 0.4% for ferritin, zinc, calcium, VA, and VD, respectively. Moreover, wasting was significantly higher in girls (13.5%) than in boys (8.2%, *p* = 0.044). Stunted children had significantly lower ferritin values, while wasted children had lower VA concentrations. In contrast, VD was lower in overweight children. Lastly, this study found that height for age Z-scores (HAZ score) and Body Mass Index (BMI) for age Z-scores (BAZ score) correlated positively with ferritin, zinc, and calcium levels. **Conclusions**: Stunting, wasting, and multiple micronutrient deficiencies remain prevalent among Indonesian children under five years of age. Strengthening nutrition interventions, in particular for iron, zinc, VA, and VD, is essential to improve child growth and health outcomes in this population.

## 1. Introduction

Micronutrient deficiencies (MNDs) remain a prominent global health problem, in particular among children under 5 years of age in low- and middle-income countries (LMICs). Although efforts among governments worldwide have been made, deficiencies in essential micronutrients, such as iron, zinc, vitamin A (VA), and iodine, persist and become a global burden. For example, in Western Africa, the problem of stunting with anemia is more common than in Eastern Africa or the Caribbean. Iodine deficiency is exceptionally high in parts of Africa and Asia. Children who lack VA often do not receive enough supplementation or plant sources of carotenoids, and stunting is strongly linked to iron-deficiency anemia [1]. Moreover, a pattern of MNDs with undernutrition, notably stunting, wasting, and underweight, contributes to health and developmental outcomes.

Children under five years of age are one of the most vulnerable groups to MNDs. Anemia, as one of the most common problems, remains high in prevalence among children aged 0–4 years, as shown by the Indonesia Health Survey (SKI) 2023 at 23.08% [2], in which about half was related to iron deficiency [3]. In the past, data from the South East Asian Nutrition Survey (SEANUTS) 2012 reported that among children aged 2–5 years, 13.7% had anemia and 12.5% had iron deficiency [4], suggesting that anemia has substantially increased. Moreover, a study also suggested that deficiencies in iodine, zinc, VA, and VD are common among children under five years old [5]. MNDs affect health, growth, cognition, school achievement, productivity, recurrent infections, and even death [6].

In addition to MNDs, the double burden of malnutrition exists in Indonesia, suggesting that Indonesia may soon face the triple burden of malnutrition. In particular, Indonesia was faced with a stunting burden in the earlier decades of the 1900s, although this problem had substantially declined to 19.8% in 2024 [7]. In other LMICs, stunting was found to be a national health problem in children under five years, such as in Pakistan (40%) [8], Bangladesh (20.2%) [9], Nigeria (32%) [10], and Ethiopia (35.01%) [11].

Evidence from LMICs that have low micronutrient intake and poor dietary diversity also highlights that MNDs in young children are widespread. In Tanzania, for example, VD deficiency was observed in 60% of breastfed infants at two weeks, and half of them had VD insufficiency at three months [12]. In Ethiopia, 62.1% of children aged 6–23 months did not receive the minimum recommended micronutrient intakes, with only 27.6% achieving adequate intake [13]. In the Amhara region, the risk of insufficient intake was very high for VA (67.6%), B-vitamins (B1: 71.6%, B3: 91.5%, B6: 95.8%, B12: 99.1%), vitamin C (90.1%), iron (75%), and zinc (95.3%) [14]. These conditions depict the systemic nutritional inadequacies, which are influenced by many factors such as dietary diversity, household food security, and limited access to fortified foods or supplementation.

Therefore, this study aims to examine the relationship between MNDs and nutritional status, including stunting, wasting, and overweight, among Indonesian children under five years of age, using secondary data derived from the Indonesian Basic Health Research (Riskesdas) 2018, and primary data on micronutrient levels such as iron, zinc, calcium, VA, and VD.

## 2. Materials and Methods

### 2.1. Study Area and Subjects

A cross-sectional design was conducted utilizing data from the 2018 Riskesdas study and biological specimens. Children included in this study were participants of the 2018 Riskesdas study who had stored serum specimens with adequate volume for analysis. Riskesdas employed a multistage, nationally representative sampling design involving 30,000 census blocks (CBs) and 10 households per CB. Biomedical sample collection was conducted in 1% of the total sample (3000 CBs; approximately 30,000 households). Of the 2560 children aged 0–14 years who provided blood samples, 792 were aged 0–59 months. After screening for sufficient serum volume, 550 samples from children under five years were eligible and included. Thus, a total of 550 children aged 0–59 months were included in the analysis; secondary data comprised demographic characteristics, anthropometric measurements, and nutritional status.

### 2.2. Ethical Considerations

The National Institute of Health and Research Department (NIHRD) ethical committee on 26 October 2021 (LB.02.01./2/KE/658/2021) approved this work. In addition, parents or legal guardians of participating subjects in the Riskesdas study 2018 also gave their written informed consent.

### 2.3. Nutritional Assessment

Anthropometric measurements followed standard procedures as described by the Ministry of Health of the Republic of Indonesia [15]. A multifunctional height meter with an accuracy of 0.1 cm and an electronic weighing scale (A&D, Tokyo, Japan) with an accuracy of 100 g were used to measure height and weight, respectively [16]. Nutritional status was calculated, applying height for age Z-scores (HAZ score) and Body Mass Index (BMI) for age Z-scores (BAZ score) with the World Health Organization (WHO) Anthro software version 3.2.2, 2011, and the classification of nutritional status based on the WHO standard growth chart [17]. Wasting was classified using BMI-for-age: severely wasted (BAZ < −3 SD), wasted (–3 SD < BAZ < −2 SD), normal (–2 SD < BAZ +1 SD), and overweight (+2 SD < BAZ < +3 SD), and obese (BAZ > +3 SD). Moreover, stunting was defined as a HAZ score of < −2 SD, while normal was defined as a HAZ score between ≥−2 and +1SD.

### 2.4. Serum Specimen Collection and Handling

Analysis for micronutrient values was carried out at the Research and Development Center for Biomedical and Basic Health Technology, Ministry of Health, Indonesia. The stored Riskesdas’ 2018 serum was retrieved, which was stored at −80 °C, following a standard procedure from the National Institute of Health and Research and Development. After verification, serum samples were grouped by age and sample quality (lipemic or non-lipemic). Lastly, A total of 550 eligible serum samples was used and examined for micronutrient analysis, including serum ferritin, zinc, calcium, retinol, and 25(OH)D.

### 2.5. Determination and Analysis of Serum Micronutrients

Serum samples were prepared at room temperature before analysis. VA (retinol) concentrations were determined using high-performance liquid chromatography (HPLC). Serum zinc concentrations were measured using atomic absorption spectroscopy (AAS). An enzyme-linked immunosorbent assay (ELISA) was used to measure VD, C-reactive protein (CRP), and ferritin. Lastly, an automated biochemical analyzer was used to determine the calcium level. To calculate ferritin level, although the current WHO protocol recommends using both CRP and α-1-acid glycoprotein (AGP) for inflammation adjustment [18], AGP was not measured in this study. Therefore, ferritin concentrations were adjusted using CRP alone [19], using a correction of 0.65 when CRP levels exceeded 5 mg/dL [20,21]. The deficiency cut-offs were defined according to established references: ferritin < 15 ng/mL, zinc < 0.7 mg/L, retinol < 20 μg/dL (0.70 μmol/L), VD (25-hydroxyvitamin D) < 30 ng/mL, and calcium < 8.8 mg/dL [22,23].

### 2.6. Statistical Analysis

All analyses were conducted using SPSS version 18, setting *p* < 0.05 as statistically significant. A data evaluation of the normality was performed using the Kolmogorov–Smirnov test. A descriptive analysis was executed to summarize sample characteristics and micronutrient levels. Because the primary objective of this study was to compare micronutrient biomarkers between groups rather than model determinants of nutritional status, it used univariate statistical tests (*t*-tests, chi-square tests, ANOVA). The variations in micronutrient values, HAZ, and BAZ for respondents’ gender and residence, as well as nutritional status, were also examined using a T-test. Chi-square tests were used to compare the prevalence of stunting or wasting and micronutrient status across subgroups. Furthermore, the difference in micronutrient values of different nutritional status was tested using the ANOVA test. Correlation analysis, using Pearson’s test, was assessed between the z scores of HAZ and BAZ and micronutrient values (ferritin, zinc, calcium, VA, and VD).

## 3. Results

### 3.1. Subject Characteristic and Nutrition Deficiency Prevalence

The demographic characteristics of the subjects are shown in Table 1. The majority of children lived in rural areas (64.5%), and the most common gender was boys (56.2%). Most participants were 25 to 59 months (78.2%), while the smallest proportion were aged 7–12 months (6.2%). MND profiles showed that zinc and ferritin deficiencies were the most prevalent, affecting 38.7% and 34.2% of children, respectively. In contrast, deficiencies in calcium, VA, and VD were found in a small proportion of respondents, i.e., 8.2%, 7.8%, and 0.4%, respectively.

### 3.2. Nutrition Status Regarding Gender and Residency

As shown in Table 2, the mean score HAZ indicated that both boys and girls were within the normal nutritional range (−1.25 ± 1.71 and −0.97 ± 1.75, respectively). Similarly, BAZs were normal for boys (0.21 ± 3.06) and girls (0.30 ± 3.61). The prevalence of stunting was higher in boys than in girls, with values of 25.2% and 20.3%, respectively, but this difference was not significant (*p* = 0.327). Conversely, the prevalence of wasting was significantly higher in girls (13.5%) than in boys (8.2%) (*p* = 0.044).

Table 2 also compares the means of HAZ and BAZ of children living in urban and rural areas. Both groups exhibited a normal nutritional status. A statistically significant difference was found in BAZ (*p* = 0.027), but not HAZ (*p* = 0.333), between urban and rural children. The prevalence of stunting in rural (25.6%) compared with urban areas (21.7%), and the prevalence of wasting in urban (10.6%) compared with rural areas (10.4%) were not statistically significant (*p* = 0.547 and *p* = 0.939, respectively).

### 3.3. Micronutrient Concentration Based on Nutrition Status

As summarized in Table 3, serum micronutrient levels of children aged 0–59 months with stunted status had lower ferritin levels than children with normal nutritional status (*p* = 0.038). The mean VA concentration from wasted children was 1.08 ± 0.23 μmol/L, which was lower than that of normal, possible risk, and overweight children, i.e., 1.10 ± 0.34, 1.13 ± 0.27, and 1.16 ± 0.36 μmol/L, respectively (*p* = 0.019).

VD levels in ng/mL differed significantly, showing a higher value in children at risk of overweight (68.1 ± 32.9) and overweight children (68.0 ± 34.7) compared with normal children (67.3 ± 27.3) and wasted (67.8 ± 28.3) (*p* < 0.01). Pearson’s correlation analysis in Table 4 shows a significant relationship between several micronutrient levels and nutritional status. HAZ showed a significant difference in ferritin (*p* = 0.023), zinc (*p* < 0.01), and calcium levels (*p* < 0.01). In addition, the relationship between micronutrient levels and nutritional status based on the BAZ showed significant correlations with ferritin (*p* = 0.023), zinc (*p* < 0.01), and calcium levels (*p* < 0.01). The association showed weak correlations: *r* = 0.098, 0.151, and 0.195 between HAZ and ferritin, zinc, and calcium, respectively, and *r* = 0.106, 0.150, and 0.195 between BAZ and ferritin, zinc, and calcium, respectively.

## 4. Discussion

The present study showed that most samples were from boys living in rural areas, with the majority aged 25–59 months. Importantly, no significant differences were observed in HAZ and BAZ between boys and girls, suggesting growth parameters in the study population may not be strongly influenced by sex, but rather by common underlying factors such as dietary intake, infection risk, and household environment. Similar findings were found in a previous report in school-aged children between 5 and 12 years [21]. Previous literature has suggested that boys may be biologically more vulnerable to faster growth, but the role of environmental and nutritional determinants may override sex-specific differences. For example, a study found that stunting and underweight did not differ significantly between boys and girls, although BMI-for-age occasionally showed variation [24]. This suggests that in contexts where food insecurity and limited dietary diversity prevail, both sexes are equally exposed to risks of malnutrition. Taken together, these findings highlight that nutrition interventions in Indonesia should be targeted to all children regardless of sex, with a stronger focus on addressing structural determinants such as food availability, feeding practices, and rural living conditions. Tailoring programs equally for boys and girls may be more effective and efficacious in improving child growth outcomes compared with sex-specific approaches.

This study found that the prevalence of stunting and wasting among children aged 0–59 months was 23.1% and 10.5%, respectively. Although stunting prevalence was slightly higher in boys than in girls (25.2% vs. 20.3%), the difference was not statistically significant. Conversely, wasting was significantly more prevalent among girls compared with boys (13.5% vs. 8.2%, *p* = 0.044). These findings suggest that chronic undernutrition (stunting) may be primarily driven by long-term environmental and dietary constraints that affect both sexes similarly, while acute malnutrition (wasting) could be more pronounced among girls. Similar patterns have been reported in studies from South Africa and other LMICs, where the sex gap in stunting was small, but thinness varied between boys and girls [24].

When comparing nutritional status by area of residence, this study revealed differences in BAZ but not in HAZ scores between urban and rural children. Children residing in rural areas had a lower mean BAZ score than those in urban areas, indicating that relative body mass may be more vulnerable to environmental and dietary disparities than linear growth. Interestingly, although the prevalence of stunting and wasting did not differ between urban and rural children, these patterns suggest that structural determinants of undernutrition, such as dietary inadequacy and recurrent infections, are prevalent across both urban and rural settings. Previous evidence supports similar results, showing that while rural residence is often associated with poorer dietary diversity and limited access to health services, urban poverty can present similar risks for child undernutrition [24]. These findings, collectively, highlight that interventions to reduce stunting and wasting must be inclusive across both sexes and geographic settings. Strategies focusing on improving dietary quality, preventing infections, and addressing gender-related disparities in child-feeding practices may be necessary to reduce the burden of undernutrition in this population.

This study also examined serum micronutrient concentrations in relationship to children’s nutritional status. While mean serum concentrations of zinc, calcium, VA, and VD were not significantly different between stunted and normal children, serum ferritin concentration was lower among stunted children compared with those with normal nutritional status. Iron deficiency in early life may partly play a role. Maternal iron status during pregnancy and lactation contributes to iron deficiency in early childhood. In Indonesia, the prevalence of anemia among pregnant women remains high, and this condition commonly persists during the breastfeeding period [16]. Consequently, an infant may be born with inadequate iron stores, contributing to the development of iron deficiency, which could impair growth and cognition during the first two years of life. Several studies have confirmed this intergenerational link, showing that maternal iron deficiency during pregnancy is strongly associated with lower neonatal iron stores and increased risk of stunting [25,26,27,28].

Interestingly, VA concentrations were found to be significantly higher in overweight children compared with wasted or normal peers, possibly reflecting differences in dietary quality or bioavailability of fat-soluble vitamins in children with greater body mass [29]. In addition, VD concentrations were lower in wasted children compared with those with a possible risk of overweight and overweight nutritional status. This result is similar to another study in Indian children [30]. Vitamin D concentration was lower in wasted children compared with those with possible risk of overweight and overweight nutritional status, maybe because wasting is often associated with inadequate dietary intake, poor absorption, and low body fat reserves, which reduce the storage and availability of VD. This study also highlights that other regions and countries had a prevalence of VD deficiency or insufficiency in children, yet the prevalence of deficiency was unexpectedly low (0.4%). The timing of blood collection (months and provinces sampled), local sun-exposure practices, and dietary patterns could therefore contribute to the low national prevalence observed [31,32,33,34,35,36].

In addition, this study observed a weak correlation between micronutrient levels (ferritin, zinc, and calcium) and HAZ or BAZ score. Similar results were observed in children 0–59 months in Cameroon, showing that ferritin, zinc, and calcium had a weak association against HAZ [37]. The low magnitude of statistically significant correlations, with small effect sizes, suggests limited clinical or practical relevance.

This study provides several strengths, mostly because of the use of a nationally representative dataset (Riskesdas 2018) that enhances the generalizability of the findings to Indonesian children under five years. The inclusion of biochemical indicators provides an objective assessment of micronutrient status and complements anthropometric measures, providing a picture of micronutrient and macronutrient status. However, several limitations need to be acknowledged. This study recognizes the potential for residual misclassification of the ferritin level, which was adjusted only by CRP, and not by AGP. The cross-sectional design also prevents us from drawing causal inferences about the relationship between nutritional status and biochemical outcomes. Lastly, a limitation of this study is the absence of multivariable modeling to adjust for confounders. Future studies should address limitations, such as incorporating multivariable approaches to account for interacting determinants of nutritional status and correcting ferritin levels by applying corrections based on both CRP and AGP.

## 5. Conclusions

Stunting, wasting, and multiple micronutrient deficiencies are still prevalent among Indonesian children under five years of age. Zinc and ferritin deficiencies were the most prevalent, while calcium, VA, and VD deficiencies were also present. Nutritional status was significantly associated with micronutrient profiles (ferritin, zinc, and calcium). Strengthening existing supplementation programs for adolescent girls and pregnant women is essential to prevent childhood MNDs and improve child growth outcomes.

## Figures and Tables

**Table 1:** Demographic characteristics and nutritional deficiency, as well as micronutrient deficiency of children aged 0–59 months.

Variables	*n*	(%)
Residences		
Urban	195	35.5
Rural	355	64.5
Sex		
Boys	309	56.2
Girls	241	43.8
Age group		
0–12 months	34	6.2
13–24 months	86	15.6
25–59 months	430	78.2
Micronutrient Deficiency		
Ferritin	188	34.2
Zn	213	38.7
Calcium	45	8.2
Vit A	43	7.8
Vit D	2	0.4
Nutrition Deficiency		
HAZ (Stunting)	127	23.1
BAZ (Wasting)	57	10.4

**Table 2:** The mean HAZ and BAZ scores and the nutritional status prevalence of Indonesian children aged 0–59 months by gender and residency.

Variables	*n*	Mean + SD	95% CI		*p*-Value	Nutritional Status N (%)	*p*-Value
			Lower	Upper			
Gender		HAZ				Stunting	
Boys	309	−1.25 ± 1.71	−0.57	0.01	0.737	78 (25.2%)	0.327
Girls	241	−0.97 ± 1.75	−0.57	0.01		49% (20.3%)	
Total	550	−1.11 ± 1.73	−1.27	−0.98		127 (23.1%)	
Gender		BAZ				Wasting	
Boys	309	0.21 ± 3.06	−0.65	0.48	0.343	25 (8.2%)	0.044 *
Girls	241	0.30 ± 3.61	−0.66	0.49		32 (13.5%)	
Total	550	0.25 ± 3.33	−0.27	0.53		57 (10.5%)	
Residency		HAZ				Stunting	
Urban	195	−1.10 ± 1.84	−0.19	0.41	0.333	77 (21.7%)	0.547
Rural	355	−1.20 ± 1.52	−0.17	0.40		50 (25.6%)	
Total	550	−1.11 ± 1.73	−1.27	−0.98		127 (23.1%)	
Residency		BAZ				Wasting	
Urban	195	0.41 ± 3.92	−0.65	0.48	0.027 *	37 (10.6%)	0.939
Rural	355	−0.04 ± 1.69	−0.66	0.49		20 (10.4%)	
Total	550	0.25 ± 3.33	−0.27	0.53		57 (10.5%)	

HAZ, height-for-age Z-Score; BAZ, BMI-for-age Z-score. * Indicates a significant value of <0.05.

**Table 3:** Mean (±SD) concentrations of ferritin, zinc, calcium, vitamin A, and vitamin D across categories of height-for-age (HAZ) and BMI-for-age (BAZ) nutritional status of children 0–59 months in Indonesia.

Nutrition Status	Ferritin (ng/mL) Mean ± SD	*p*-Value	Zinc (mg/L) Mean ± SD	*p*-Value	Calcium (mg/dL) Mean ± SD	*p*-Value	Vitamin A (μmol/L) Mean ± SD	*p*-Value	Vitamin D (ng/mL) Mean ± SD	*p*-Value
HAZ										
Stunting (*n* = 127)	27.2 ± 25.1	0.038 *	0.73 ± 0.23	0.255	10.1 ± 0.83	0.368	1.06 ± 0.31	0.546	67.7 ± 25.8	0.108
Normal (*n* = 423)	28.1 ± 27.6		0.75 ± 0.22		9.96 ± 1.16		1.12 ± 0.33		67.4 ± 29.6	
Total (*n* = 550)	27.9 ± 27.0		0.74 ± 0.22		9.99 ± 1.10		1.11 ± 0.32		67.5 ± 28.8	
BAZ										
Wasted (*n* = 57)	28.4 ± 30.5	0.449	0.74 ± 0.21	0.176	9.80 ± 1.65	0.134	1.08 ± 0.23	0.019 *	67.8 ± 28.3	<0.01 **
Normal (*n* = 388)	28.5 ± 27.0		0.73 ± 0.23		10.0 ± 1.06		1.10 ± 0.34		67.3 ± 27.3	
Possible risk of overweight (*n* = 59)	26.6 ± 24.0		0.79 ± 0.25		10.2 ± 1.07		1.13 ± 0.27		68.1 ± 32.9	
Overweight (*n* = 46)	24.6 ± 28.1		0.74 ± 0.20		9.91 ± 0.84		1.16 ± 0.36		68.0 ± 34.7	
Total (550)	27.9 ± 27.0		0.74 ± 0.22		9.99 ± 1.10		1.11 ± 0.32		67.5 ± 28.8	

HAZ, height-for-age Z-score; BAZ, BMI-for-age Z-score. * Indicates a significant value of < 0.05 and ** indicates a significant value of <0.01.

**Table 4:** Correlations between micronutrient concentrations and nutritional status (HAZ and BAZ) in 0-to-59-month-old Indonesian children.

Micronutrients	HAZ	BAZ
Ferritin (ng/mL)	0.098 * (*p* = 0.023)	0.106 * (*p* = 0.023)
Zn (mg/L)	0.151 ** (*p* < 0.01)	0.150 ** (*p* < 0.01)
Calcium (mg/dL)	0.195 ** (*p* < 0.01)	0.195 ** (*p* < 0.01)
Vitamin A (μmol/L)	−0.018 (*p* = 0.667)	0.035 (*p* = 0.420)
Vitamin D (ng/mL)	−0.019 (*p* = 0.653)	−0.720 (*p* = 0.093)

* Indicates a significant *p* value of < 0.05 and ** indicates a significant *p* value of < 0.01.

## Data Availability

The data that support the findings of this study are not openly available due to the Indonesian Ministry of Health policy.

## Abbreviations

The following abbreviations are used in this manuscript:

MNDs	Micronutrient deficiencies
LMICs	Low- and middle-income countries
VA	Vitamin A
VD	Vitamin D
SEANUTS	Southeast Asian nutrition survey
SKI	Indonesia health survey
Riskesdas	Indonesian basic health research
NIHRD	National Institute of Health and Research Department
HAZ score	Height for age Z-scores
BAZ score	Body Mass Index (BMI) for age Z-scores
WHO	World Health Organization
HPLC	High-performance liquid chromatography (HPLC)
AAS	Atomic absorption spectroscopy
ELISA	Enzyme-linked immunosorbent assay
CRP	C-reactive protein
AGP	α-1-acid glycoprotein
